# Directed differentiation into insulin-producing cells using microRNA manipulation

**DOI:** 10.1515/med-2020-0170

**Published:** 2020-06-19

**Authors:** Michael D. Williams, Mugdha V. Joglekar, Anandwardhan A. Hardikar, Wilson K. M. Wong

**Affiliations:** Diabetes and Islet biology Group, NHMRC Clinical Trials Centre, Faculty of Medicine and Health, The University of Sydney, Level 6, Medical Foundation Building, 92-94 Parramatta Road, Camperdown, NSW 2050, Australia

**Keywords:** microRNA, beta-cells, islets, differentiation, non-coding RNA, type 1 diabetes

## Abstract

Our commentary is focused on three studies that used microRNA overexpression methods for directed differentiation of stem cells into insulin-producing cells. Islet transplantation is the only cell-based therapy used to treat type 1 diabetes mellitus. However, due to the scarcity of cadaveric donors and limited availability of good quality and quantity of islets for transplant, alternate sources of insulin-producing cells are being studied and used by researchers. This commentary provides an overview of distinct studies focused on manipulating microRNA expression to optimize differentiation of embryonic stem cells or induced pluripotent stem cells into insulin-producing cells. These studies have used different approaches to overexpress microRNAs that are highly abundant in human islets (such as miR-375 and miR-7) in their differentiation protocol to achieve better differentiation into functional islet beta (β)-cells.

Type 1 diabetes (T1D) mellitus is characterized by immune-mediated destruction of insulin-producing β-cells in the pancreatic islets [1]. The management plan for T1D consists of exogenous insulin injections or continuous glucose monitors with/without insulin pumps. Although effective, these treatment options fail to completely recapitulate the true biology of a healthy pancreas to control real-time fluctuations in circulating glucose. Currently, this can be achieved only through total pancreas or islet transplantation, which replace the lost insulin-producing β-cells. Islet transplantation has developed over the years to be the only cell-based therapy to treat T1D. In 2000, Shapiro et al. [2] described a procedure for the transplantation of allogeneic islets from the human cadaveric pancreas (The Edmonton Protocol) in recipients with T1D. This study demonstrated that patients with T1D can achieve independence from exogenous insulin administration with excellent metabolic control when glucocorticoid-free immunosuppression is combined with the infusion of an adequate islet mass. While the results are very promising, this technique will continue to be limited by low numbers of cadaveric donors and the restricted yield of good quality and quantity of islets for transplantation. This has led researchers to pursue alternative cell sources with the potential to differentiate into insulin-producing cells for transplantation [3,4]. This targeted differentiation has been achieved via multiple different protocols including the application of growth factors and the use of small molecules as signaling inducers as well as recapitulating stepwise process of differentiation of embryonic definitive endoderm into fully differentiated β-cells. In this commentary, we describe three studies where microRNAs (miRs) are used for direct differentiation of stem cells toward β-cells.

In pursuit of an alternative source of islet cells for transplantation, Lahmy et al. [5] chose to explore the differentiation capacity of the human-induced pluripotent stem cells (hiPSCs) into an insulin-producing phenotype utilizing lentiviral-mediated overexpression of a single miR. MiRs are small, noncoding RNA molecules that are now recognized as one of the regulators of gene expression, fine-tuning several pathological and physiological processes. The authors presented a novel strategy to generate insulin-producing cells *in vitro* using miR-375 overexpression in the absence of additional extrinsic factors. MiR-375 is known to be one of the most abundant miRs in human islets and is involved in regulating insulin secretion, β-cell development, and proliferation [6]. The authors generated hiPSC lines from human foreskin fibroblasts and then transduced those hiPSCs using lentiviral particles containing miR-375 (pCDH-375) and noncoding controls (pCDH-NCs) at a multiplicity of infection (MOI) of 25. Differentiated miR-375 overexpressing cells reorganized themselves into small bud-like aggregates, demonstrated dual immune-positive labels for insulin and C-peptide, and glucose-responsive insulin secretion following a standard glucose challenge protocol. Cells transduced to overexpress miR-375 had higher transcript levels of β-cell-related genes, including *HNF4α*, *PDX1*, *NKX6.1*, and *PAX6*, suggesting that miR-375 upregulation alone could facilitate the differentiation of hiPSCs into an islet-like phenotype. The authors also demonstrated that overexpression of miR-375 can induce directed differentiation of human embryonic stem cells (hESCs) into insulin-producing pancreatic lineage [7].

Similar to miR-375, miR-7 is also one of the most abundant endocrine miRs in rodents [8] and human islets [9]. In a recent study, Lopez-Beas et al. [10] overexpressed miR-7 in (HS181) hESCs and differentiated them into a β-cell-like phenotype with an established multistep differentiation protocol [11]. The authors initially performed a microarray analysis to detect differentially expressed miRs in hESCs (that are undifferentiated, differentiated, or spontaneously differentiated), human islets, as well as human pancreas. They confirmed that miR-7 is the most abundant miR in human islets. The miR expression analysis during different stages of differentiation of hESCs into insulin-producing β-like cells was performed using the real-time polymerase chain reaction (PCR). MiR-7 was found to be abundant at very early stages of differentiation with a gradual reduction in the expression at later stages of directed differentiation. The fluorescence *in situ* hybridization (FISH) method also validated miR-7 expression to be high across differentiating hESCs in the early stages and reducing later. The authors observed that the β-like cells obtained after directed differentiation of hESCs with their protocol do not have high expression of miR-7 as is seen in the human islets. Therefore, they extrinsically increased miR-7 expression during the differentiation process to understand if that would enhance pancreatic differentiation of hESCs.

A scrambled control and miR-7 mimic were transiently transfected into the differentiating hESCs at day 14 of the multistep differentiation protocol and were incubated for 24 h in the differentiation medium. A 100 nM concentration of miR-7 mimic was found to be optimum with a significant increase in the miR-7 levels as well as an increase in insulin transcripts. The expression of pancreatic transcription factors, *FOXA*2 and *PDX1*, was also significantly increased in miR-7 mimic-transfected cells compared to that in the controls.

To examine the insulin secretion potential, differentiated cells were incubated under different glucose conditions (2 and 20 mM) with or without the membrane depolarizing agent potassium chloride (KCl; 30 mM). The miR-7 mimic transfected cells demonstrated increased insulin secretion in response to the increasing glucose concentration compared to scramble/mock control and anti-miR-7 transfected cells. Double immunofluorescence and MetaMorph-based quantitative analysis also confirmed that higher levels of insulin and C-peptide were observed after the differentiation of miR-7 mimic transfected hESCs.

The aforementioned studies [5,7,10] presented a novel strategy to generate insulin-producing cells *in vitro* by miR overexpression. Immunocytochemistry, gene expression profile, and glucose-stimulated insulin secretion assays confirmed that miR overexpressing cells share characteristics with those of mature islets. These studies illustrate the potential of miR manipulations on directed differentiation of stem cells into endocrine pancreatic lineage.

To generate more β-cells for transplantation, many groups around the world have explored methods to reliably induce the differentiation of progenitor or stem cell populations into endocrine pancreatic cells, using a variety of techniques. These include the use of growth/differentiation factors in islet-derived cells [12] or stepwise application of small molecules in embryonic stem cells [13,14]. An alternative to culture-based methods to induce differentiation is direct gene expression intervention. When *Pdx1*, *Ngn3* (also referred to as *Neurog3*), and *MafA* are reexpressed in an experimental context, these three primary factors have been shown to induce the differentiation of exocrine pancreatic acinar cells into functional insulin-producing cells, indistinguishable from endogenous islet β-cells [15]. Unfortunately, currently, there are no small molecules or pharmaceuticals capable of inducing the expression of *Pdx1* or *Ngn3*, rendering genetic manipulation as the only available technique.

Lahmy et al. [5,7] and Lopez-Beas et al. [10] have explored an alternative to direct transcription factor manipulation. These studies have demonstrated that the overexpression of miR (miR-375 or miR-7) has the potential to drive the differentiation process to endocrine pancreatic lineage. It will be interesting to understand the combined effect of overexpression of multiple β-cell-specific miRs in the differentiation of stem/progenitor cells. The miRs associated with pancreas development, biology, and disease have been investigated and thoroughly reviewed [16,17], offering a wealth of potential miRs for overexpression experiments. The success of these studies represents a proof of concept application for miR-based approaches to alter cell identity in cell replacement therapies. This technique could also be applied to a variety of other cell types, disease states, and clinical applications.

Stem cell differentiation to generate β-cells suitable for clinical transplantation to treat T1D has been studied for more than a decade and has shown substantial progress over the years. It still faces many challenges and requires more optimization and refinement. Current protocols [13,14] can be further fine-tuned by using regulatable miRs/other noncoding RNAs or miR inhibitors at specific stages during the differentiation process to closely resemble a true β-cell (Figure 1). Identification of an approach to generate β-cells *in vitro* would highly benefit people with T1D by making β-cell transplantation readily available as a treatment.

**Figure 1:**
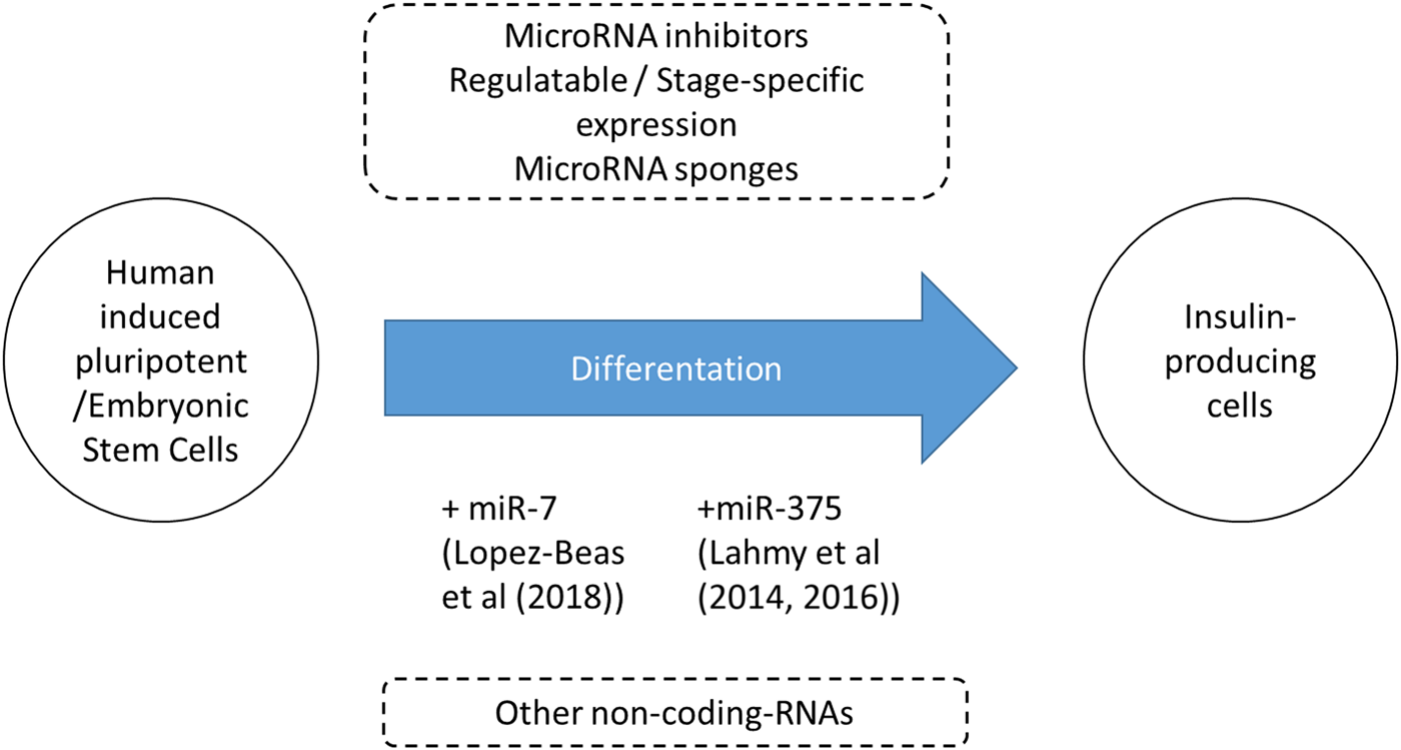
This commentary describes distinct studies (by Lopez-Beas et al and Lahmy et al) that used miR-7 and miR-375 for directed differentiation of stem cells into insulin-producing cells respectively. While innovative, other approaches (in dashed boxes) such as miR inhibitors or other noncoding RNAs can also be used in the future to enhance the β-cell differentiation process.
